# O Antigen Allows *B. parapertussis* to Evade *B. pertussis* Vaccine–Induced Immunity by Blocking Binding and Functions of Cross-Reactive Antibodies

**DOI:** 10.1371/journal.pone.0006989

**Published:** 2009-09-14

**Authors:** Xuqing Zhang, Maria Eugenia Rodríguez, Eric T. Harvill

**Affiliations:** 1 Department of Veterinary and Biomedical Sciences, Pennsylvania State University, University Park, Pennsylvania, United States of America; 2 Department of Chemistry, Centre of Applied Biotechnology (CINDEFI, CCyT La Plata), School of Science, La Plata University, La Plata, Argentina; Hannover School of Medicine, Germany

## Abstract

Although the prevalence of *Bordetella parapertussis* varies dramatically among studies in different populations with different vaccination regimens, there is broad agreement that whooping cough vaccines, composed only of *B. pertussis* antigens, provide little if any protection against *B. parapertussis*. In C57BL/6 mice, a *B. pertussis* whole-cell vaccine (wP) provided modest protection against *B. parapertussis*, which was dependent on IFN-γ. The wP was much more protective against an isogenic *B. parapertussis* strain lacking O-antigen than its wild-type counterpart. O-antigen inhibited binding of wP–induced antibodies to *B. parapertussis*, as well as antibody-mediated opsonophagocytosis *in vitro* and clearance *in vivo*. aP–induced antibodies also bound better *in vitro* to the O-antigen mutant than to wild-type *B. parapertussis*, but aP failed to confer protection against wild-type or O antigen–deficient *B. parapertussis* in mice. Interestingly, *B. parapertussis*–specific antibodies provided in addition to either wP or aP were sufficient to very rapidly reduce *B. parapertussis* numbers in mouse lungs. This study identifies a mechanism by which one pathogen escapes immunity induced by vaccination against a closely related pathogen and may explain why *B. parapertussis* prevalence varies substantially between populations with different vaccination strategies.

## Introduction

Whooping cough is an acute, highly contagious, paroxysmal coughing illness [1]. The first whooping cough vaccines consisting of whole inactivated *B. pertussis* were licensed in the mid-1940s and led to a dramatic decrease of disease incidence [1], [2]. However, the potential health risk associated with whole cell vaccines led to the development of acellular vaccines, consisting of some combination of *B. pertussis* antigens including pertussis toxin (PT), pertactin, filamentous hemaglutinin (FHA) and 2 fimbriae serotypes. Despite maintenance of high vaccine coverage, the reported whooping cough incidence has been increasing over the past 20 years in some developed countries [3], [4], although a large portion of whooping cough infections are thought to remain unreported [5]. Both *B. pertussis* and *B. parapertussis* are causative agents of whooping cough [1], [6] that appear to have evolved independently from distinct lineages of *B. bronchiseptica* through rearrangements and large scale gene loss, with *B. parapertussis* emerging more recently than *B. pertussis* [7], [8]. Although they are closely related, a few striking differences exist between the human-adapted bordetellae. For example, *B. parapertussis* lipopolysaccharide (LPS) includes a repetitive membrane-distal O-antigenic structure, while *B. pertussis* only expresses lipid A and a branched-chain core oligosaccharide with a complex trisaccharide modification, but lacks O-antigen [9], [10]. *B. pertussis* expresses PT, but *B. parapertussis* does not due to mutations in the promoter region [11], [12]. Since differential diagnosis of *B. pertussis* and *B. parapertussis* does not affect the course of treatment, it is rarely performed in clinical settings [13], [14]. The CDC does not list *B. parapertussis* as reportable [3], but a few epidemiological studies have reported the percentage of whooping cough cases caused by *B. parapertussis* to be from 1% to 98%, most commonly 4–40% [13]. Although *B. parapertussis* appears to contribute substantially to disease, whooping cough vaccines are solely derived from *B. pertussis* [1].

Clinical and experimental data indicate that whooping cough vaccines are very efficacious against *B. pertussis* but not against *B. parapertussis* [14]–[18], however, a mechanistic understanding of this phenomenon has not been described. While whooping cough vaccines may fail to generate efficient cross-immunity against *B. parapertussis*, it is also possible that cross-reacting adaptive immunity is generated but is evaded by *B. parapertussis*. Recently, our lab showed that the O-antigen of *B. parapertussis* shields it from *B. pertussis*-infection-induced antibodies [19], relevant to the natural immune-mediated competition between *B. pertussis* and *B. parapertussis* in unvaccinated population. However, nearly all people in industrialized countries are vaccinated, changing the immune landscape of the host population and the immune-mediated competition between these two human pathogens.

To examine the mechanisms used by *B. parapertussis* to evade *B. pertussis* vaccines-induced immunity, we showed that a *B. pertussis* whole cell vaccine (wP) had some effect, but a commercial acellular vaccine (aP) had no effect against *B. parapertussis* growth in mouse lungs. IFN-γ contributes to the protection against *B. parapertussis* by wP. O-antigen shielded *B. parapertussis* from the binding of vaccine-induced antibodies, interfered with opsonophagocytosis of *B. parapertussis* mediated by aP and wP-induced antibodies and blocked antibody-mediated clearance *in vivo*. wP conferred more protection against an isogenic *B. parapertussis* strain lacking O-antigen, indicating that O-antigen contributed to the evasion of wP-induced immunity. aP, however, failed to induce cross-protection against *B. parapertussis* with or without the hindrance of O-antigen. In *B. pertussis* vaccinated hosts, supplement of *B. parapertussis*-specific, but not *B. pertussis*-specific, antibodies conferred protection against *B. parapertussis*, indicating that the lack of proper antibody responses causes the failure of these vaccines against *B. parapertussis*. Together these results explain the clinical finding that *B. parapertussis* avoids clearance by the current vaccines, and provides a mechanistic understanding that will guide new approaches to overcoming this problem.

## Materials and Methods

### Bacterial strains and growth

*B. pertussis* strain 536, *B. parapertussis* strain CN2591 and its isogenic mutant lacking O-antigen, CN2591Δ*wbm*, have been described previously [10], [20]. For opsonization, attachment and phagocytosis experiments, these strains were transformed with plasmid pCW505 (kindly supplied by Dr. Alison Weiss, Cincinnati, Ohio) which induces cytoplasmic expression of GFP without affecting growth, or antigen expression [21]. Bacteria were maintained on Bordet-Gengou agar (Difco) supplemented with 10% sheep's blood (Hema Resources) and 20 µg/mL streptomycin (Sigma-Aldrich). Liquid cultures were grown overnight in Stainer-Scholte broth at 37°C to mid-log phase [22], [23].

### Cells

Peripheral blood polymorphonuclear leukocytes (PMNs) were isolated from heparinized venous blood using Ficoll-Histopaque (Sigma, St Louis, MO) gradient centrifugation. PMNs were harvested and the remaining erythrocytes were removed by hypotonic lysis. Cell viability was >99% as determined by Trypan Blue exclusion. Prior to functional assays, PMNs were washed twice with Dulbecco's modified Eagle medium (DMEM) (Hyclone) supplemented with 10% of fetal calf serum (FCS) (Hyclone), resuspended, and used immediately. All experiments were carried out with freshly isolated PMNs lacking FcγRI (CD64) expression, as monitored by FACS analysis using a fluorescence-activated cell sorter FACScan (Becton Dickinson, San Jose, CA) with anti-FcγRI mAb 22 [24].

### Opsonization

GFP-expressing strains were opsonized by incubation at 37°C with 5% heat-inactivated wP-induced/naive or aP/adjuvant-induced serum samples for 30 min in a final volume of 50 µL. Serum opsonized bacteria were incubated with R-phycoerythrin (RPE)–labeled goat F(ab′)_2_ fragments of anti-mouse IgG (Southern Biotechnology, Birmingham, AL) for 30 min at 4°C. Opsonization of each *Bordetella* strain was determined by FACS analysis [25].

### Attachment and phagocytosis

Attachment and phagocytosis of the *Bordetella* strains were evaluated as previously described with a few modifications [26]. Briefly, serum opsonized GFP-expressing bacteria were incubated with PMNs at multiplicity of infection (MOI) of 30 for 20 min at 37°C to allow binding. After extensive washing to remove non-attached bacteria, an aliquot was maintained on ice to be used for bacterial attachment control. Another aliquot was further incubated for 1 h at 37°C to allow internalization. Phagocytosis was stopped by placing PMNs on ice. Cell surface bound bacteria in both aliquots (before and after 1 hour incubation at 37°C) were detected by incubation with RPE–labeled goat F(ab′)_2_ fragments of anti-mouse IgG at 4°C for 30 min. To avoid eventual nonspecific binding of antibodies, all incubations were done in the presence of 25% heat-inactivated human serum. After washing, samples were analyzed by flow cytometry. Ten thousand cells were analyzed per sample. Green fluorescence intensity associated with PMNs maintained at 37°C for 20 min has previously been shown to represent bacterial attachment [25]. Phagocytosis was calculated from the drop in mean red fluorescence intensity of green-positive cells after incubation for additional1h at 37°C as described [25].

### Animal experiments

C57BL/6 mice were obtained from Jackson Laboratories (Bar Harbor) and bred in our *Bordetella*-free, specific pathogen-free breeding rooms at The Pennsylvania State University. 4–6 week old mice were sedated with 5% isoflurane (Abbott Laboratory) in oxygen and vaccinated by intraperitoneally (i.p.) injection of 1×10^8^ CFU of heat-inactivated bacteria in 1 mL of phosphate balanced saline (PBS, Omnipur) (wP), 1/5 human dose of Adacel (Sanofi Pastuer) (0.5 µg PT, 1 µg FHA, 0.6 µg pertactin, 5 µg fimbriae 2 and 3 per mouse) with Imject Alum (Thermo Scientific) (aP) or only Imject Alum in 200 µL PBS on day 14 and 28 prior to challenge [27]. For challenge, mice were sedated and inoculated by pipetting 50 µL PBS containing 5×10^5^ CFU of the indicated bacteria onto the external nares [28]. This method reliably distributes the bacteria throughout the respiratory tract [29]. For adoptive transfer of immune serum, mice were vaccinated with the indicated bacteria on day 0 and 14 and sera were collected on day 28 or sera were collected from naïve animals. 200 µL of sera were i.p. injected at the time of inoculation [30], [31]. For quantification of bacteria numbers, mice were sacrificed via CO_2_ inhalation and the lung, trachea, and nasal cavity were excised. Tissues were homogenized in PBS, serial diluted and plated onto Bordet-Gengou agar plates with 20 µg/mL streptomycin, and colonies were counted after incubation at 37°C for 3–5 days [30]. Gamma interferon (IFN-γ) was depleted by i.p. injections of 5 mg of the antibody from hybridoma XMG1.2 one day prior to challenge [32]. The lower limit of detection was 10 CFU. For all experiments, protocols were reviewed and approved by the Pennsylvania State University IACUC and all animal were handled in accordance with institutional guidelines.

### Splenocyte re-stimulations

Spleens were excised from groups of 3–4 C57BL/6 mice after vaccination. Splenocytes were isolated as previously described [27], [33]. In brief, spleens were homogenized and red blood cells were lysed with 0.84% ammonium chloride. 2×10^6^ cells were re-suspended in DMEM supplemented with 10% FCS, 1 mM sodium pyruvate (HyClone), and 100 µg/mL penicillin and streptocycin (HyClone) and placed into each well of a 96-well tissue culture plate. Splenocytes were stimulated with either media alone or media containing 10^7^ CFU (MOI of 5) of the indicated bacteria that had been heat-killed [27], [33]. After three days, the supernatants were collected and analyzed for IFN-γ and interleukin-10 (IL-10) production via Enzyme-linked immunosorbent assays (ELISA) as per the manufacturers' instructions (R&D Systems).

### Titer ELISAs

Antibody titers were determined as previously described [19], [34], [35]. Briefly, heat-inactivated or exponential phase live bacteria were diluted to 5×10^7^ CFU/mL in a 1∶1 mix of 0.2 M sodium carbonate and 0.2 M sodium bicarbonate buffers. These antigens were coated onto 96-well plates, incubated for 2 h at 37°C in a humidified chamber, washed and blocked. A1∶50 or 1∶10 dilution of wP-induced/naive or aP/adjuvant-induced serum samples from an individual mouse was added to the first well of each row and serially diluted 1∶2 across the plates. Plates were incubated for 2 h at 37°C, washed and probed with 1∶4000 dilution of goat anti-mouse Ig horseradish peroxidase (HRP)-conjugated antibodies (Southern Biotech) for 1 h and visualized with 2,2′-Azino-bis (3-ethylbenzothiazoline-6-sulfonic acid) diammonium salt in phosphate-citrate buffer with hydrogen peroxide at an absorbance of 405 nm. Titers were determined via the endpoint method based on optimal density of identically treated wells probed with naïve or adjuvant-induced sera.

### Western blot analysis

Lysates containing 5×10^5^ CFU of indicated heat-killed bacteria were run on 7% sodium dodecyl sulfate-polyacrylamide gel electrophoresis (SDS-PAGE) gels in denaturing conditions. Polyvinylidene Fluoride (PVDF) membranes (Millipore**)** were probed overnight with either naïve serum or serum from vaccinated mice at a 1∶10 or 1∶100 dilution for aP and wP-induced serum respectively. 1∶10,000 dilution of goat anti-mouse Ig HRP-conjugated antibodies (Southern Biotech) was used as the detector antibody [19], [36]. The membrane was visualized with ECL Western Blotting Detection Reagent (Pierce Biotechnology).

### Statistical analysis

The means +/− standard error (error bars) were determined for all appropriate data. Two-tailed, unpaired Student's T-tests were used to determine statistical significance between groups. Results were also analyzed by ANOVA and Tukey simultaneous test in Minitab with similar significance. All experiments were performed at least twice with similar results.

## Results

### *B. parapertussis* O-antigen contributes to the evasion of wP–induced immunity

**Figure 1:**
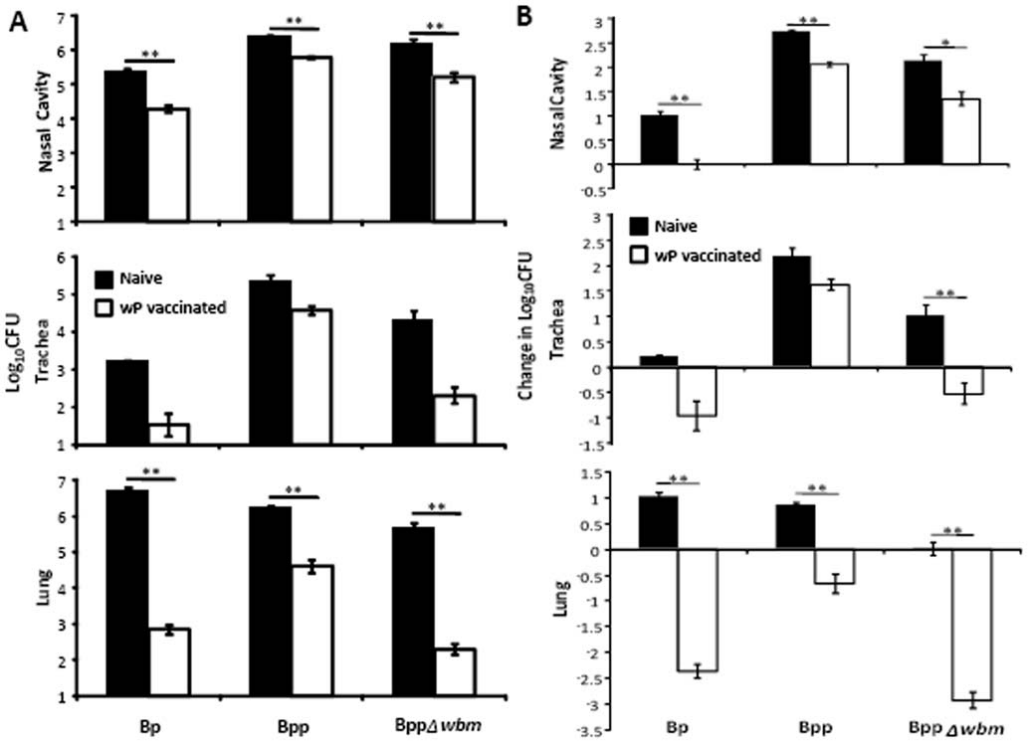
*B. parapertussis* is more susceptible to wP–induced immunity in the absence of O-antigen. Groups of four naïve (black) or wP vaccinated (white) C57BL/6 mice were challenged with the indicated bacteria. (A) The number of CFUs recovered from the respiratory tract on day 3 post-challenge is expressed as the Log_10_ mean ± the standard error. Decreases in Log_10_CFU in vaccinated mice compared to naïve mice on day 3 post-challenge are indicated underneath the *x* axes. (B) The change in CFU number over the first 3 days after challenge is expressed as change in Log_10_ mean ± the standard error. * indicates P≤0.05. ** indicates P≤0.01. The limit of detection is indicated as the lower limit of the *y* axes.

To examine whether wP is cross-protective against *B. parapertussis* and whether O-antigen interferes with its cross-protection, naïve or wP vaccinated C57BL/6 mice were challenged with 5×10^5^ CFU of *B. pertussis, B. parapertussis* or an isogenic *B. parapertussis* strain lacking O-antigen (BppΔ*wbm*). In comparison to naïve mice, wP treatment reduced *B. pertussis* numbers by 91.9%, 97.8% and >99.9% in the nasal cavity, trachea and lungs by day 3 post challenge; naïve mice having about 7000 fold more bacteria in the lungs than vaccinated mice (Figure 1A). wP vaccination reduced *B. parapertussis* loads by 76.6%, 83.0% and 97.6% in the nasal cavity, trachea and lung; naïve mice having about 40 fold more bacteria in the lungs than vaccinated mice (Figure 1A). These results are consistent with multiple clinical studies showing whole cell vaccines confer good protection against *B. pertussis* but relatively little protection against *B. parapertussis* [16], [17], [37]. Interestingly, compared to naïve mice, wP reduced numbers of an O-antigen deficient *B. parapertussis* strain by 89.4%, 99% and >99.9% in the nasal cavity, trachea and lung; naïve mice having around 2000 fold more bacteria in the lungs than vaccinated mice (Figure 1A). The fold protection (reduction of bacterial number in each individual vaccinated mouse compared to mean number of bacteria in naive mice on day 3 post-challenge) of wP against O-antigen deficient *B. parapertussis* was significantly higher than against wild-type *B. parapertussis* in both trachea and lung (lung: P = 0.0006, trachea: P = 0.018), indicating that wP is more efficacious against O-antigen deficient *B. parapertussis*.

To understand how vaccination affects the infection, it is important to examine these effects in the context of the dynamic infectious process. *B. pertussis* and *B. parapertussis* increased in numbers throughout the respiratory tract of naïve mice over 3 days, reflecting effective colonization and bacterial growth (Figure 1B). O-antigen deficient *B. parapertussis* grew in the nasal cavity and trachea but not in the lungs of naïve mice due to its increased susceptibility to complement [38] (Figure 1B). wP decreased the numbers of *B. pertussis* in all respiratory organs over 3 days, resulting in a net decline in numbers, particularly in the lower respiratory tract (LRT). Vaccination did not decrease *B. parapertussis* numbers as efficiently in the lung and *B. parapertussis* actually grew in numbers in the trachea and nasal cavity, reflecting successful colonization and expansion despite vaccination (Figure 1B). Interestingly, wP vaccination decreased O-antigen deficient *B. parapertussis* numbers as efficiently as it did *B. pertussis* numbers in the trachea and lung (Figure 1B). Together these results are consistent with clinical studies showing that wP vaccination confers relatively little protection against *B. parapertussis* and show that O-antigen is required for *B. parapertussis* to avoid efficient wP vaccine-induced immunity.

**Figure 2:**
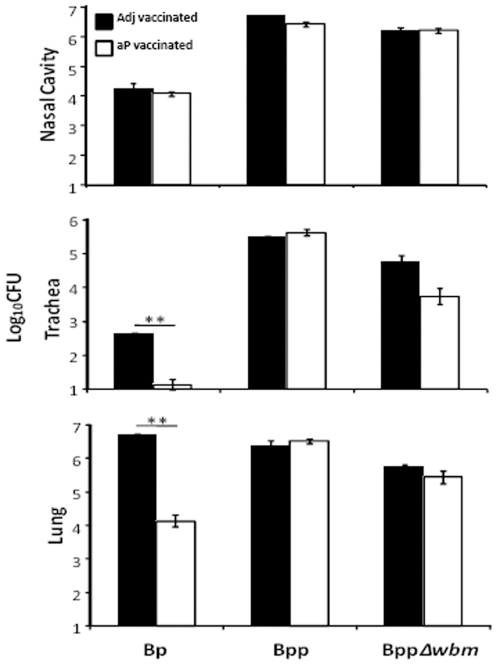
aP does not reduce *B. parapertussis* numbers. Groups of four C57BL/6 mice were vaccinated with adjuvant only (black) or aP (white), challenged with the indicated bacteria and sacrificed on day 3 post-challenge. The numbers of CFUs recovered from the respiratory tract are expressed as the Log_10_ mean ± the standard error. ** indicates P≤0.01. The limit of detection is indicated as the lower limit of the *y* axes.

Many developed countries have switched to acellular vaccines, although these provide even less protection against *B. parapertussis* [16], [18], [37]. We therefore determined if the O-antigen of *B. parapertussis* contributes to the evasion of aP-induced immunity. While aP reduced *B. pertussis* colonization in both lung and trachea of vaccinated mice by 99.7% and 96.8% compared to the mice given just adjuvant, aP had no effects on either *B. parapertussis* or O-antigen deficient *B. parapertussis* colonization (Figure 2). These data indicate that aP does not induce protective immunity against *B. parapertussis.*

### wP induces T cells that cross react with *B. parapertussis*

**Figure 3:**
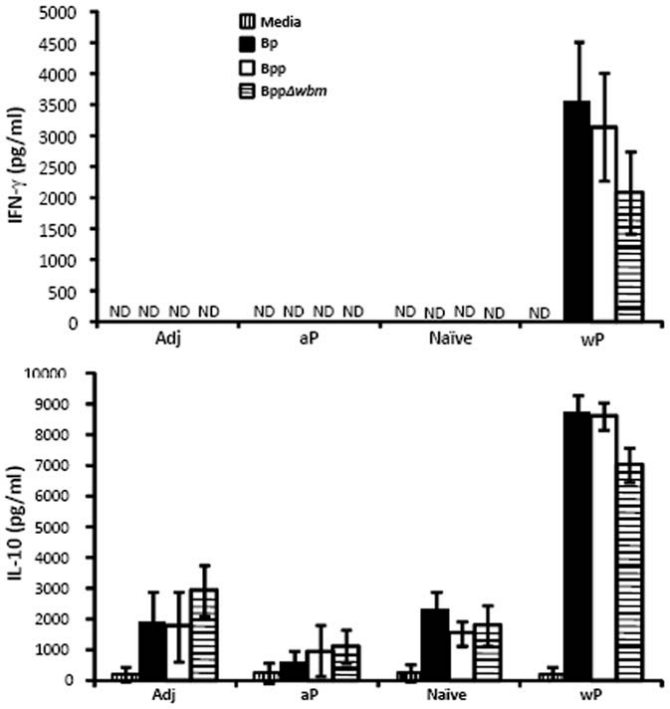
Splenic production of IFN-γ and IL-10 is cross-reactive. Splenocytes from groups of four naïve C57BL/6 mice or mice vaccinated with the indicated vaccine were stimulated with media only (vertically hatched), heat-killed *B. pertussis* (black), *B. parapertussis* (white) or O-antigen deficient *B. parapertussis* (horizontally hatched) for 3 days. The concentration of IFN-γ and IL-10 in the supernatant is expressed as the mean ± the standard error. ND indicates none detected.

To investigate why aP is less effective than wP against *B. parapertussis* and why wP confers different levels of protection against *B. pertussis*, O-antigen deficient and wild-type *B. parapertussis*, we compared their induction of T cell responses known to be important for control and clearance of *B. parapertussis* [34]. Splenocytes from mice that were naïve, vaccinated with wP, aP or adjuvant were stimulated with heat-killed *B. pertussis* or wild-type or O-antigen deficient *B. parapertussis*. After 3 days, IFN-γ and IL-10 production, representative of T_H_1 and T_H_2 responses, was measured. aP vaccination did not affect IFN-γ or IL-10 levels, which were similar to those induced by naïve and adjuvant-treated controls (Figure 3). In contrast, splenocytes from wP-vaccinated mice responded similarly to heat-killed *B. parapertussis* or *B. pertussis*, producing significantly higher IFN-γ and IL-10 than splenocytes from naïve or adjuvant-treated mice (Figure 3). IFN-γ and IL-10 production was abolished in TCRβ/δ^−/−^ mice (data not shown), indicating that vaccine-induced IFN-γ and IL-10 production is dependent on T cells. These data indicate that the T-cell response to wP is cross-reactive to *B. parapertussis* and that O-antigen did not affect the T cell response.

### IFN-γ contributes to wP–induced protection against *B. parapertussis*

**Figure 4:**
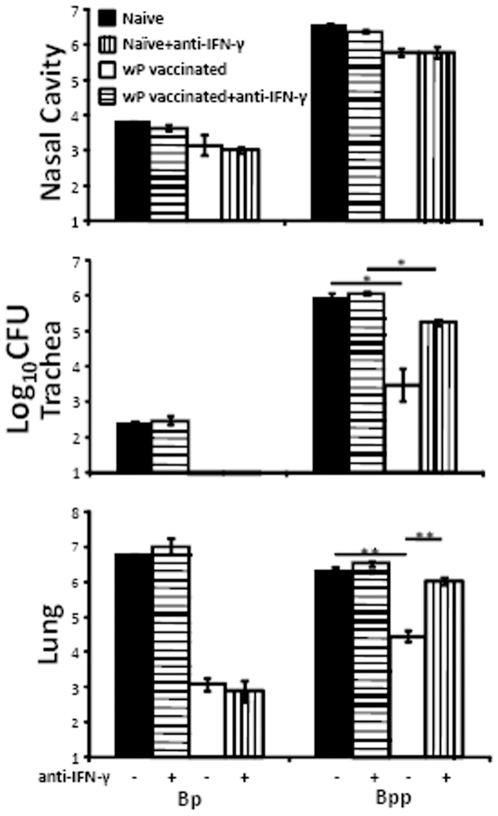
IFN-γ contributes to the protection against *B. parapertussis* by wP. Groups of four naïve (black and horizontally hatched) or wP vaccinated (white and vertically hatched) C57BL/6 mice were untreated (−) (black and white) or i.p. injected with (+) (horizontally and vertically hatched) anti-IFN-γ antibody, challenged with *B. parapertussis* and sacrificed 3 days post-challenge. The number of CFUs throughout the respiratory tract is expressed as the Log_10_ mean ± the standard error. * indicates P≤0.05. ** indicates P≤0.01. The limit of detection is indicated as the lower limit of the *y* axes.

In the vaccination studies above (Figure 1, Figure 2), protection against *B. parapertussis* correlates with the high IFN-γ responses of wP but not aP vaccinated animals (Figure 3). IFN-γ has been shown to contribute to leukocyte recruitment and the reduction of bacterial numbers during *B. parapertussis* infection (D.N. Wolfe, A.T. Karanikas, S.E. Hester, M.J. Kennett, E.T. Harvill, submitted for publication). To determine how the cross-reactive IFN-γ response after wP vaccination might contribute to its protection against *B. parapertussis*, naïve or wP vaccinated mice were left untreated or depleted of IFN-γ, challenged with *B. pertussis* or *B. parapertussis* and sacrificed 3 days later for bacterial enumeration. Vaccination or IFN-γ depletion had no effects on colonization of the nasal cavity (Figure 4). However, *B. pertussis* numbers were reduced by >99.9% and >99.5% in the lung and trachea of vaccinated mice compared to naïve mice regardless of the presence or absence of IFN-γ (Figure 4). Although wP reduced *B. parapertussis* numbers in the lung and trachea by about 98.6% and 99.6%, this effect was abolished in mice given IFN-γ neutralizing antibodies (Figure 4), indicating that IFN-γ contributes to the protection conferred by wP against *B. parapertussis*.

### Serum antibody responses to aP and wP are cross-reactive to denatured but not live *B. parapertussis*

**Figure 5:**
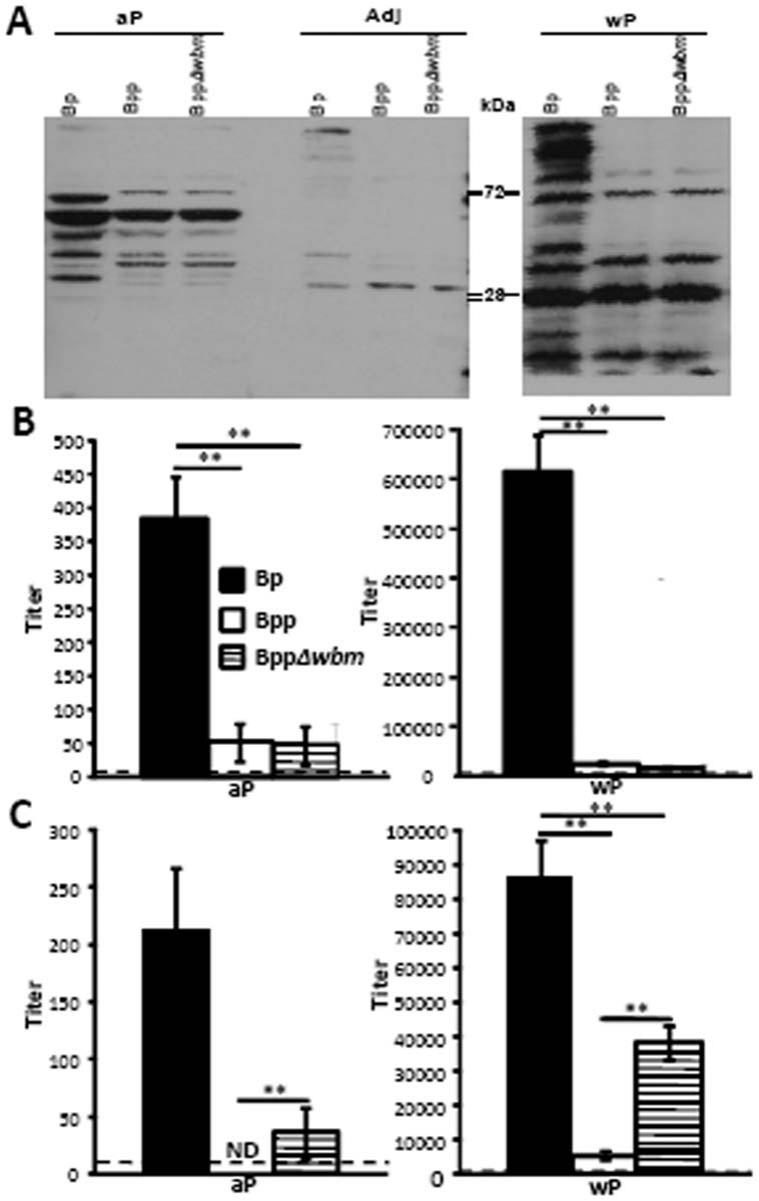
O-antigen inhibits the binding of *B. pertussis* vaccine-induced antibodies to live, but not denatured, *B. parapertussis* cells. (A) Western blots were performed on lysates of indicated bacteria probed with serum antibodies collected from mice that were vaccinated with aP, adjuvant only (Adj) or wP. (B) Heat-inactivated or (C) live *B. pertussis* (black), *B. parapertussis* (white) or O-antigen deficient *B. parapertussis* (hatched) were coated on ELISA plate. Serum antibody titer of mice vaccinated with aP or wP is expressed as mean of the end point titers of four independent samples ± the standard error. ** indicates P≤0.01. The dashed line indicates the limit of detection.

We have previously shown that antibodies are required for anamnestic immunity to *B. parapertussis* [34]. To determine whether *B. pertussis* vaccines induce antibodies that recognize and/or protect against *B. parapertussis*, we first tested whether antibody responses are cross-reactive between strains and if O-antigen affects the responsiveness of antibodies. In a western blot, whole cell extracts of *B. pertussis*, wild-type and O-antigen deficient *B. parapertussis* were probed with serum antibodies from aP, adjuvant only, wP vaccinated or naïve mice. Compared to the control, aP vaccination induced serum antibodies that recognized distinct antigens present in all three bacteria, although the size and intensity of bands appeared to differ between *B. pertussis* and *B. parapertussis*. While wP-induced serum antibodies recognized four major bands in denatured *B. parapertussis*, they recognize more antigens in *B. pertussis*, especially those of high molecular weights (Figure 5A).

To quantify the cross-reactivity of antibodies, we determined the titers of aP or wP-induced serum antibodies by ELISA using heat-inactivated bacteria as antigens. wP-induced sera had much higher titers than aP-induced sera (Figure 5B). For both aP and wP-induced serum antibodies, *B. pertussis*-specific antibody titers were much higher than *B. parapertussis*-specific antibody titers regardless of the presence or absence of O-antigen (Figure 5B). Since heat killing releases many antigens that are not exposed on the surface of live bacteria, similar experiments were performed with live bacteria as antigens. Interestingly, both wP and aP-induced serum antibodies bound to live *B. parapertussis* more efficiently in the absence of O-antigen (Figure 5C). These data suggest that O-antigen interferes with the binding of *B. pertussis* vaccine-induced antibodies to live *B. parapertussis*.

### O-antigen inhibits aP– and wP–induced, antibody-mediated opsonization and subsequent attachment to, and phagocytosis by, PMNs

**Figure 6:**
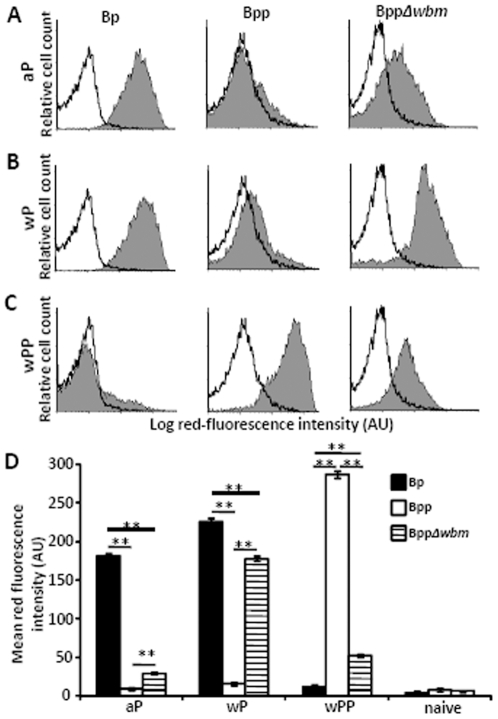
O-antigen decreases the opsonization of *B. parapertussis* by *B. pertussis* vaccine-induced antibodies. Representative histograms show flow cytometric analysis of indicated bacteria opsonized with naïve serum (white) or (A) aP, (B) wP, (C) wPP-induced serum (grey) and stained with RPE-labeled goat F(ab)_2_ fragments of anti-mouse IgG. (D) Mean red-fluorescence of *B. pertussis* (black), *B. parapertussis* (white), O-antigen deficient *B. parapertussis* (hatched) opsonized with indicated serum from four individual mice ± the standard error is shown. AU indicates arbitrary units. ** indicates P≤0.01.

To determine whether O-antigen interferes with key functions of antibodies, we assessed its effects on opsonization and subsequent attachment to and phagocytosis by PMNs mediated by aP or wP induced antibodies. Consistent with the ELISA results (Figure 5C), *B. pertussis* was efficiently opsonized by both aP and wP-induced serum antibodies (Figure 6A, 6B, and 6D). Although *B. parapertussis* was efficiently opsonized by whole cell *B. parapertussis* (wPP)-induced serum antibodies (Figure 6C and 6D), aP or wP-induced antibodies failed to opsonize this bacterium (Figure 6A, 6B, and 6D). In contrast, O-antigen deficient *B. parapertussis* was efficiently opsonized by aP or wP-induced antibodies (Figure 6A, 6B, and 6D), indicating that O-antigen hinders the opsonization of *B. parapertussis* by both aP and wP-induced serum antibodies.

**Figure 7:**
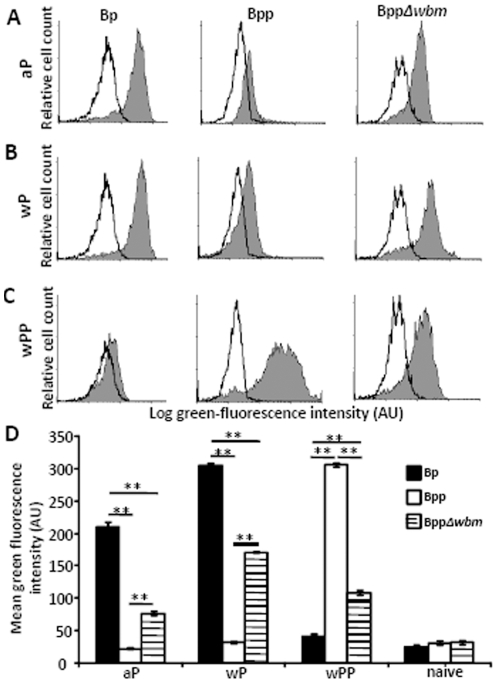
O-antigen blocks *B. pertussis* vaccine-induced antibodies from mediating adherence of *B. parapertussis* to PMNs. Naïve serum (white) or (A) aP, (B) wP, (C) wPP-induced serum (grey)-opsonized GFP-expressing bacteria were incubated with freshly isolated human peripheral blood PMNs. Representative histograms of flow cytometry analysis of these cells are shown. (D) Mean green fluorescence associated with PMNs incubated with GFP-expressing *B. pertussis* (black), *B. parapertussis* (white) or O-antigen deficient *B. parapertussis* (hatched) opsonized with four independent indicated serum ± the standard error is shown. AU indicates arbitrary units. ** indicates P≤0.01.

To examine if O-antigen blocking of opsonization results in inhibitory effects on attachment of *B. parapertussis* to phagocytes, bacteria opsonized with vaccine-induced antibodies were further incubated with polymorphonuclear leukocytes (PMN) for 20 mins and cell surface bound bacteria numbers were identified by flow cytometry. aP or wP-induced-antibody-opsonized *B. pertussis* attached to PMNs efficiently (Figure 7A, 7B, and 7D). *B. parapertussis* attached to PMN after opsonization with wPP-induced serum antibodies (Figure 7C and 7D), but neither aP nor wP-induced antibodies mediated attachment of this bacterium to PMNs (Figure 7A, 7B, and 7D). *B. parapertussis* lacking O-antigen, opsonized with aP or wP-induced antibodies, efficiently attached to phagocytes (Figure 7A, 7B, and 7D), indicating that O-antigen also inhibits this process.

**Figure 8:**
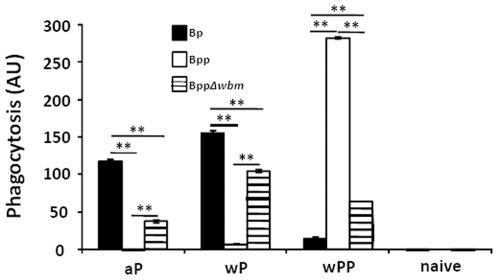
O-antigen blocks *B. pertussis* vaccine-induced, antibody-mediated phagocytosis of *B. parapertussis*. Freshly isolated human peripheral blood PMNs were incubated for 20 min or 1 h and 20 min with GFP-expressing *B. pertussis* (black), *B. parapertussis* (white), or O-antigen deficient *B. parapertussis* (hatched), that are opsonized with serum from aP, wP, wPP-vaccinated or naïve mice. The cell surface bound bacteria were detected by incubation with RPE-labeled goat F(ab')_2_ fragments of anti-mouse IgG. Mean phagocytosis calculated from the decrease in red-fluorescence of green-positive cells incubated for 1 h and 20 min compared to that incubated for 20 min of experiments done with 4 independent serum samples ± the standard error was shown. AU indicates arbitrary units. ** indicates P≤0.01.

To determine if O-antigen interferes with aP or wP-induced antibodies ability to mediate phagocytosis of *B. parapertussis* by PMNs, an aliquot of cells from the attachment experiment was incubated for one extra hour and phagocytosis was measured. No significant internalization of naïve serum-opsonized bacteria was observed. *B. pertussis* opsonized with aP or wP-induced antibodies was efficiently phagocytosed, but similarly treated *B. parapertussis* was not (Figure 8). In contrast, wPP-induced antibody-opsonized *B. parapertussis* was efficiently internalized by PMNs (Figure 8). *B. parapertussis* lacking O-antigen opsonized by aP or wP-induced antibodies was efficiently phagocytosed by PMNs. Together these results indicate that O-antigen protects *B. parapertussis* from aP or wP-induced serum antibody mediated opsonization, attachment to phagocytes and subsequent internalization by these cells.

### O-antigen blocks antibody-mediated clearance of *B. parapertussis* from mouse lungs

**Figure 9:**
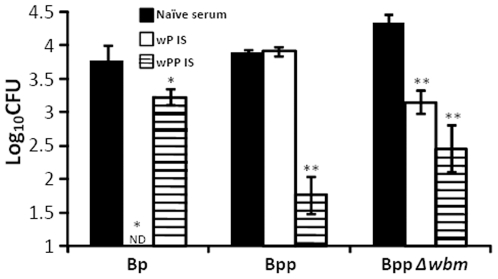
Passive transfer of wP–induced serum antibodies mediates clearance of O-antigen deficient, but not wild-type, *B. parapertussis* from mouse lungs. Groups of four C57BL/6 mice were adoptively transferred naïve serum (black), wP-induced serum (white) or wPP-induced serum (hatched) at the time of bacterial challenge and dissected 14 days later. The number of CFUs recovered from lung is expressed as the Log_10_ mean ± the standard error. * indicates P≤0.05 and ** indicates P≤0.01 compared to mice given naïve serum. The limit of detection is indicated as the lower limit of the *y* axes. ND indicates undetectable bacterial number.

Since O-antigen interferes with the binding of *B. pertussis* vaccine-induced antibodies to live *B. parapertussis* and phagocytosis dependent on these antibodies *in vitro*, we tested if O-antigen inhibits antibody-mediated clearance *in vivo*. Serum from naïve (NS), wP- or wPP-vaccinated mice were transferred to naïve animals. Bacterial loads in the lungs were determined on day 14 post *B. parapertussis* challenge since antibodies have no effect until *B. parapertussis* specific T cell responses are generated around day 14 [30], [39] (D.N. Wolfe unpublished data). Serum from wPP vaccinated animals significantly lowered numbers of both wild-type and O-antigen deficient *B. parapertussis* compared to naïve serum treated animals and modestly reduced *B. pertussis* numbers (Figure 9). This indicates that although lower level of phagocytosis of O-antigen deficient *B. parapertussis* mediated by wPP-induced serum antibodies were observed compared to wild-type strain (Figure 8), this low level of phagocytosis and/or other biological activities of passively transferred antibodies might be sufficient to reduce O-antigen deficient strain *in vivo* (Figure 9). wP-induced serum completely cleared *B. pertussis* from the lung by day 14 post-challenge but did not reduce *B. parapertussis* numbers. These serum antibodies did, however, reduce the numbers of O-antigen deficient *B. parapertussis* by more than 90% (Figure 9), indicating that O-antigen prevents the wP-induced serum antibody mediated reduction of *B. parapertussis* numbers *in vivo*.

### *B. parapertussis*–specific antibodies augment *B. pertussis* vaccine-induced protective immunity against *B. parapertussis*

**Figure 10:**
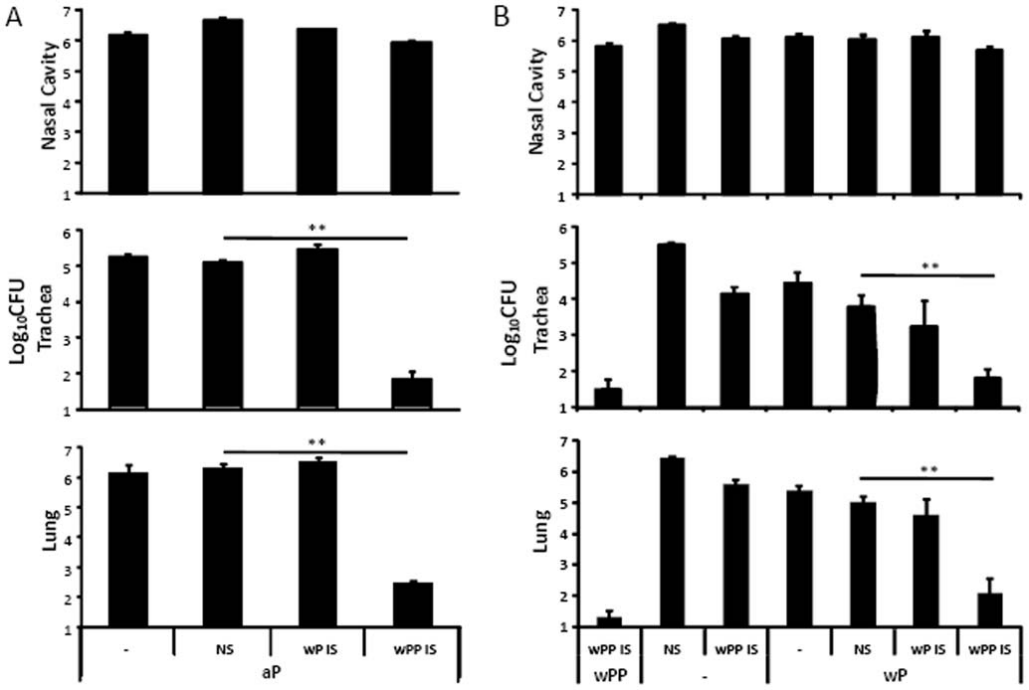
Passive transfer of *B. parapertussis*–specific antibodies rapidly reduces *B. parapertussis* colonization in aP and wP vaccinated animals. Groups of four C57BL/6 mice were (B) left untreated (−) or vaccinated with (A) aP, (B) wPP or wP. Mice lacking a transfer of antibodies (−) or given naïve serum (NS), wP-induced serum (wP IS) or wPP-induced serum (wPP IS) were challenged with *B. parapertussis*. Mice were sacrificed three days post-challenge and the number of CFUs recovered from the respiratory tract is expressed as the Log_10_ mean ± the standard error. ** indicates P≤0.01. The limit of detection is indicated as the lower limit of the *y* axes.

We have previously shown that both antibodies and T cells are required for anamnestic protective immunity against *B. parapertussis* [34]. Although wP vaccination induced T cell responses that were cross-reactive (Figure 4) and antibodies that bound antigens from heat-killed *B. parapertussis* (Figure 5B), O-antigen decreased the binding of these antibodies to live *B. parapertussis* (Figure 5), the opsonophagocytosis mediated by these antibodies *in vitro* (Figure 6, Figure 7, Figure 8) and their antibody-mediated clearance *in vivo* (Figure 9). Based on these observations, we hypothesized that wP induces sufficient T cell response but the antibody response is not sufficient to clear *B. parapertussis* because O-antigen protects *B. parapertussis* from wP-induced antibodies. If this were the case, then adding antibodies that bind live *B. parapertussis* should render wP induced immunity sufficient to rapidly clear *B. parapertussis*. To test this, mice were vaccinated with aP (Figure 10A) or wP (Figure 10B), these vaccinated mice were left untreated or given naïve, wP or wPP-induced serum antibodies at the time of *B. parapertussis* challenge and sacrificed three days after challenge. To compare the protective immunity to that generated by *B. parapertussis*, a group of mice were vaccinated with wPP and adoptively transferred with wPP-induced antibodies. Less than 100 CFUs of *B. parapertussis* were recovered in the LRT of this group of mice by day 3 post-challenge (Figure 10B). The very high titers of this wPP-induced serum antibodies alone had modest effect on reducing *B. parapertussis* numbers (Figure 10B), consistent with prior results with convalescent serum [30], [39]. In both aP and wP vaccinated mice, naïve sera had no effect, indicating that components in the serum other than those induced by vaccination do not affect bacteria numbers. Transfer of wP-induced sera had no effect on *B. parapertussis* colonization throughout the respiratory tract. However, *B. parapertussis* numbers were significantly lower in the LRT of both aP and wP vaccinated animals given wPP-induced sera than in those given naïve sera (Figure 10). These data suggest that the addition of *B. parapertussis* specific antibodies is sufficient to render wP and aP effective against *B. parapertussis*.

## Discussion

The increasing incidence of whooping cough in highly vaccinated developed countries includes an unknown proportion of *B. parapertussis* infections[3], [4]. Multiple clinical and experimental studies have shown that current whooping cough vaccines have little efficacy against this bacterium [14]–[18], but have not revealed why. In this study, we determined that O-antigen shields vaccine-induced antibodies from binding to *B. parapertussis* and prevents antibody-mediated opsonophagocytosis *in vitro* and antibody-mediated clearance *in vivo*. Although O-antigen requires a large multigenic *wbm* locus to produce and *B. pertussis* is still successful in the human population without this surface antigen [40], it is retained in *B. parapertussis* [7]. Apart from its role in inhibiting complement deposition and complement-mediated killing [38], our study suggests that O-antigen, by decreasing *B. pertussis*-vaccine-induced antibody binding, may confer a selective advantage to *B. parapertussis* in human populations in which there is high prevalence of detectable immunity to *B. pertussis*.

It is interesting that wP, but not aP, induced IFN-γ, which contributed to protection against *B. parapertussis* but not *B. pertussis* (Figure 4). IFN-γ plays a role in the recruitment and activation of phagocytic cells [41], [42]. In *B. parapertussis* infected mice, the peak of neutrophil numbers in the lung on day 7 and subsequent control of *B. parapertussis* numbers are dependent on IFN-γ (D.N. Wolfe, A.T. Karanikas, S.E. Hester, M.J. Kennett, E.T. Harvill, submitted for publication). *B. pertussis* challenged IFN-γ^−/−^ mice, however, have an indistinguishable course of infection in the respiratory tract and recruit similar numbers of leukocytes to the site of infection as compared to wild-type mice [43] (Wolfe unpublished data), indicating that IFN-γ is not required for *B. pertussis* clearance and leukocyte recruitment. In vaccinated IFN-γR^−/−^ mice, no impaired reduction of *B. pertussis* numbers is observed during the first week after challenge, although a rebound of bacteria number was observed on day 10 [44]. This is consistent with our observation that IFN-γ is not required for vaccine-mediated control of *B. pertussis* numbers on day 3 post-challenge (Figure 4).

Consistent with multiple clinical and experimental studies, we found that while wP confers some level of protection against *B. parapertussis,* aP does not [16], [18]. The decrease of pertussis acellular vaccines efficacy against *B. parapertussis* compared to whole cell vaccines has been suggested to be attributable to the failure of antibodies induced by pertussis acellular vaccines to block the adherence of *B. parapertussis* to epithelial cells [45] or the immune suppressive effects of the FHA included in those vaccines [46]. Among the antigens included in the aP, only fimbriae, but not FHA and pertactin, was shown to confer cross-protection against *B. parapertussis* in a mouse model [37], [47]. wP contains more antigens than aP, among which there may be cross-protective antigens. Alternatively, the differences in Th1/Th2 skewing of wP and aP may affect vaccine efficacy. wP induces a relatively balanced Th1/Th2 response whereas aP induces a Th2-type response [48]. Our data showed that wP, but not aP, induced high splenic IFN-γ production and wP was no longer protective against *B. parapertussis* when IFN-γ was depleted, suggesting that IFN-γ contributes to the protection conferred by wP (Figure 3, Figure 4). These data are therefore consistent with the idea that inducing a Th1 response enhances immunity against *B. parapertussis*. The different Th1/Th2 skewing, quantity and quality of cross-reactive antibodies and possible immune suppression factors in aP may also explain why aP vaccination was not sufficient to induce protection against *B. parapertussis* even without the hindrance by O-antigen.

Our data reveal an interesting paradox. wP-mediated clearance of *B. parapertussis* by day 3 requires IFN-γ, which aP does not induce (Figure 3, Figure 4). Yet when wPP-induced antibodies, which had limited effect in naïve animals in the first week of infection (Figure 10B) [30], were given to aP-vaccinated animals, *B. parapertussis* was effectively cleared from mouse lungs within 3 days (Figure 10A). IFN-γ appears to contribute to the protection conferred by wP, but aP-vaccination contributes to rapid clearance despite the lack of detectable IFN-γ induction in our splenocyte re-stimulation assay (Figure 3). It is possible that some low level of IFN-γ was induced by aP vaccination, which we failed to detect, or some other cross-reactive protective T cell cytokine responses aid in the rapid clearance mediated by *B. parapertussis*-specific antibodies in aP-vaccinated animals.

Although it is not clear how much *B. parapertussis* contributes to the resurgence of whooping cough, the low efficacy of current vaccines against this bacterium might confer a selective advantage to *B. parapertussis*, relative to *B. pertussis*. Moreover, the lower protection against *B. parapertussis* conferred by acellular vaccines than whole cell vaccine could affect the relative prevalence, a possibility of greater significance since the recent switch from whole cell to acellular vaccines and the even more recent introduction of acellular vaccines, including the one used here, for adolescents and adults [16], [18], [49], [50]. Considering adults to be a possible reservoir for transmission to infants [51], [52], the lack of cross-protection of Adacel, determined in this study, may open a niche for *B. parapertussis* not only in adolescents/adults but also in infants. This study shows that *B. parapertussis* evades *B. pertussis* vaccine induced immunity by blocking cross-reactive antibodies binding via O-antigen. Our current data provide strong evidence that including more *B. pertussis* proteins that induce antibodies that recognize orthologs in *B. parapertusis* is unlikely to improve vaccines so that they protect against *B. parapertussis*. Since our study indicates that supplementing wP or aP with *B. parapertussis*-specific antibodies rendered them effective against *B. parapertussis* (Figure 10), addition of protective antigens of *B. parapertussis* to the vaccine may substantially improve its efficacy against this pathogen.
